# Meiosis-associated expression patterns during starvation-induced cell fusion in the protist *Fisculla terrestris*

**DOI:** 10.1186/s12915-025-02246-3

**Published:** 2025-05-22

**Authors:** Shan Gao, Marcel Dominik Solbach, Jens Bast, Kenneth Dumack

**Affiliations:** Institute for Zoology, University of Cologne, Zuelpicher Str. 47b, Cologne, 50674 Germany

**Keywords:** Meiosis, Protist, Rhizaria, Differentially expressed genes (DEGs), Meiosis toolkits, Cell fusion, Recombination

## Abstract

**Background:**

Unicellular eukaryotes were widely considered to r eproduce without sex. However, recent findings suggest that meiosis, and by extension (sometimes cryptic) sexual reproduction, might be present in almost all eukaryotic lineages.

**Results:**

Here, we investigate the transcriptomic response underlying starvation-induced fusion in the Rhizaria protist *Fisculla terrestris*. Investigations of differentially expressed genes (DEGs) with a particular focus on the expression of meiosis-associated genes suggest that some form of meiosis and recombination might occur in these Rhizaria.

**Conclusions:**

We showed that starvation triggered changes in gene expression of meiosis-associated genes in *F. terrestris*. However, if these processes are coupled with sexual reproduction remains to be investigated.

**Supplementary Information:**

The online version contains supplementary material available at 10.1186/s12915-025-02246-3.

## Background

**Fig. 1:**
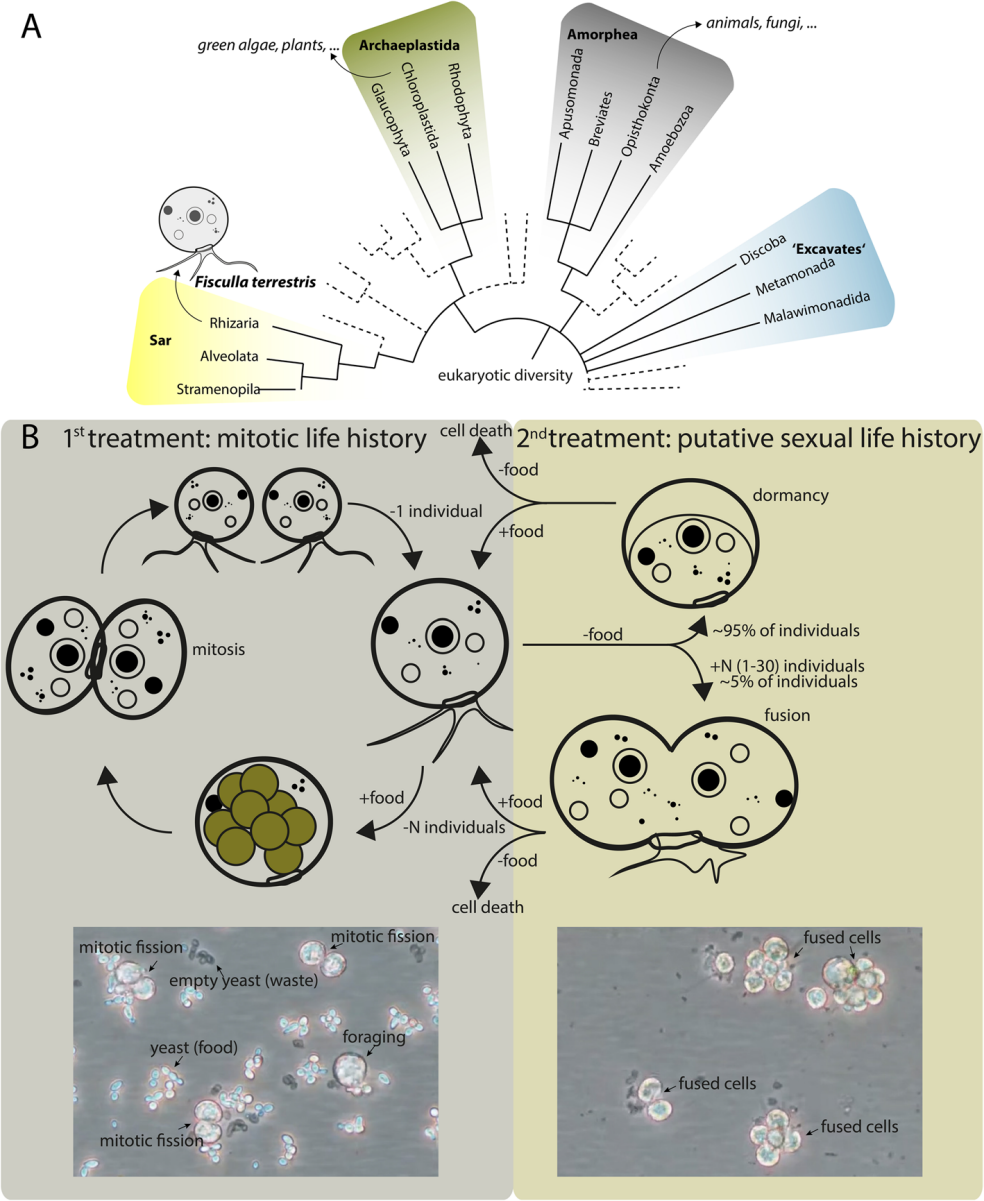
Evolutionary relationship and life history of the used organism. **A** *F. terrestris* is a representative of the Rhizaria, being only distantly related to the well-known eukaryotes like plants (Archaeplastida) and animals or fungi (Amorphea). The phylogenetic tree is based on Burki et al. [8] and only the most important eukaryotic groups are indicated by names; other branches are reduced. **B** The life history of *F. terrestris* is divided into the two treatments used for the differential genes expression (DGE) experiment, i.e., predominantly mitotically growing, non-starved individuals (left, i.e., the non-starved treatment) and predominantly starved individuals comprising dormant and fused individuals (right, i.e., the starved treatment)

Meiotic sex, the reciprocal genetic exchange between individuals, including the fusion of nuclei, recombination, and segregation of chromosomes, was commonly assumed to be restricted almost entirely to animals, fungi, and plants [1]. The vast majority of unicellular eukaryotes, i.e., protists, were considered to be asexual; however, recent findings suggest the existence of some form of sex. Animals, fungi (both Amorphea), and plants (Archaeplastida) are unrelated lineages nesting in the large diversity of eukaryotes (Fig. 1) [2]. The apparent polyphyly of sexual macroorganisms and the conserved manner of eukaryotic sex (via meiosis) suggest an early evolutionary origin of sexual reproduction within eukaryotes. Intriguing is the conservancy and homology of eukaryotic meiosis-associated (including “Meiosis-specific genes” and “Meiosis-related genes” from the meiosis toolkit) enzymes to recombination enzyme machinery of prokaryotes [3–5] and the finding of archaeal cells that undergo fusion coupled with recombination, a process being very similar to eukaryotic sex [6]. It is therefore commonly suggested that sex evolved with the eukaryotic common ancestor (that derived in some form from an archaeal ancestor) [1, 7]. Nonetheless, it is important to note that there is considerable ascertainment bias in our understanding of sex across eukaryotic lineages, as many protistan groups remain understudied.

While sexual reproduction has been well-documented in plants, animals, and fungi, there is growing evidence for its occurrence across a wide range of eukaryotic lineages [9]. This evidence comprises observations of the fusion of cells, the fusion of nuclei (karyogamy), certain chromosomal behaviors that were interpreted as meiosis, and subsequent fission [10–12]. Although fusion in protists is widely observed, the entire sequence of putative sexual behaviors could so far only be described in very few taxa. Accordingly, it proved difficult to gather direct evidence for sex in protists [13] and attention of the research field shifted to the accumulation of indirect evidence. Currently, the most common approach to investigate sex in protists is thus screening for genes that are considered to be involved in meiosis [5, 14–17]. With this, diverse groups of protists have been shown to possess meiosis-associated genes [4, 16–18]. Since these genes seem to be conserved in almost all eukaryotic lineages, it was concluded that sex must be widely distributed, cryptic, and not necessarily coupled with offspring production [19–21]. However, recently, it was questioned to rely solely on the detection of these meiosis-associated genes as evidence for sex, as these genes possibly exert pleiotropic functions [22, 23].

The Thecofilosea (Rhizaria, protists) are only very distantly related to the well-known sexual and macroscopic eukaryotes (Fig. 1A). Thecofilosea species can undergo a complete life history under laboratory conditions, including fusion, karyogamy (fusion of nuclei), and fission [11, 24]. *Fisculla terrestris* (Thecofilosea) is a unicellular, shell-bearing amoeba living in soils where it feeds predominantly on fungi (yeasts and spores) and algae. In *Fisculla terrestris*, the depletion of prey reliably triggers the fusion of cells and karyogamy, a subsequent addition of prey (or its supernatant) induces the separation of fused individuals (Fig. 1B). This process seems reminiscent of “cyclical asexuality,” i.e., the ability to switch to sexual life cycles under unfavorable conditions, while otherwise reproducing asexually [25, 26]. To generate insights into potentially occurring meiosis and sex in Thecofilosea, we identify the expression response to starvation-induced fusion in *Fisculla terrestris* with a specific focus on meiosis-associated genes.

## Results

### Experimental setup and reference transcriptome assembly

In many eukaryotes, starvation triggers a switch from an asexual to a sexual reproductive cycle [27]. In the cultures used here, *F. terrestris* fed, digested, and underwent typical mitotic fission, when food (*Saccharomyces cerevisiae*) was plentiful (i.e., in the “non-starved treatment”; Fig. 1B, left). The majority of *F. terrestris* individuals entered dormancy, i.e., a state of immobility and low activity, when food was depleted (i.e., “starved treatment”). However, under starvation, about 5% of individuals fused into large aggregates with a huge, very active pseudopodial mass (Fig. 1B, right).

**Fig. 2:**
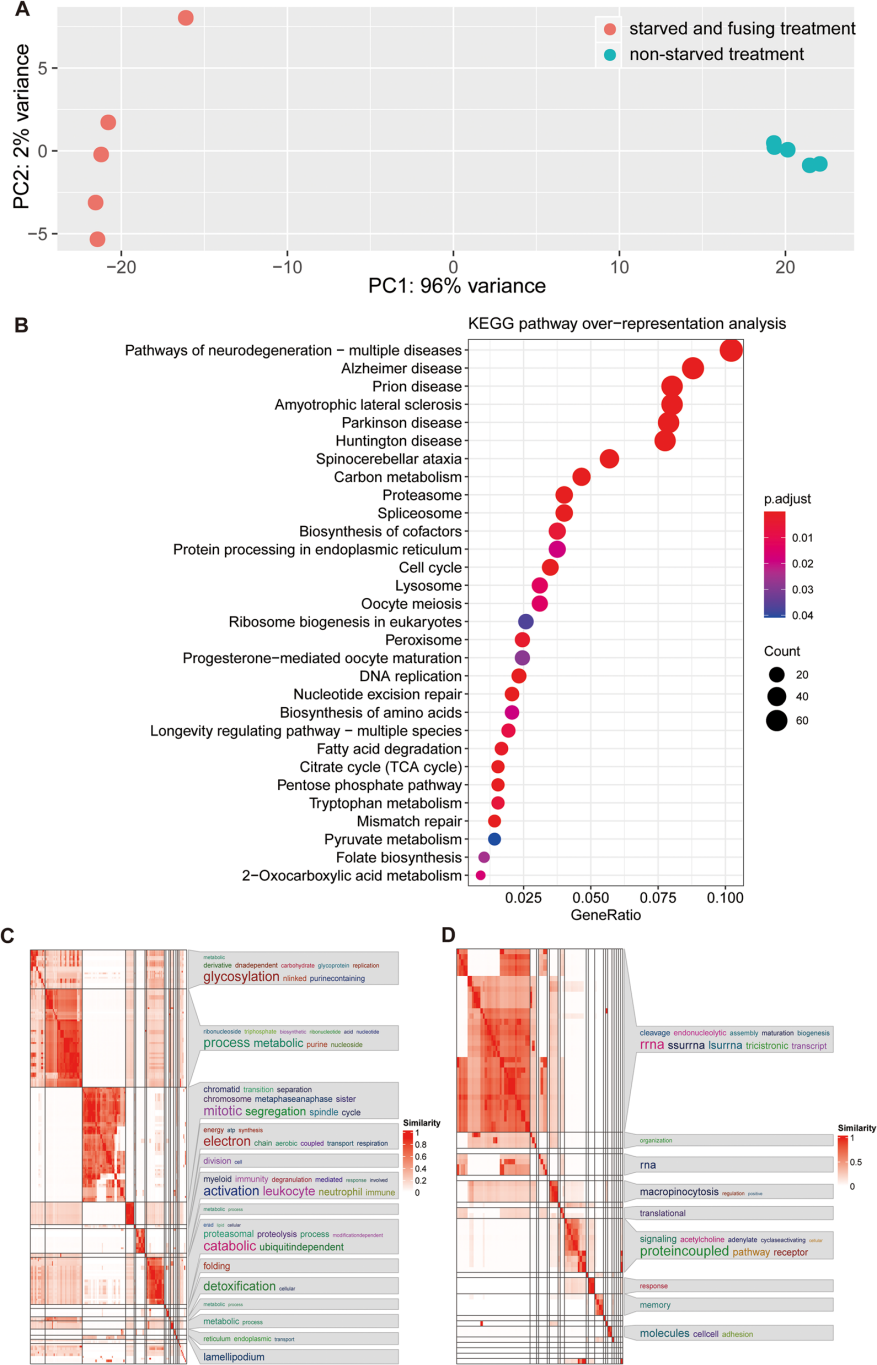
Overview of the differential gene expression data. **A** Principal component analysis (PCA) based on the expression level of all transcripts for each replicate included in the experiment. Both investigated life history stages exhibit clearly distinct expression patterns and variation within treatments was low. **B** Dot plot of KEGG pathway over-representation analysis of total DEGs. Some meiosis-associated pathways were enriched. The changed cellular functions (GO terms) in each treatment (**C** non-starved treatment, **D** starved treatment)

To identify the molecular signatures associated with fusion (and potential meiosis-facilitated sexual reproduction), we extracted RNA from five replicated cultures under each treatment, i.e., non-starved and starved. Next, we combined all data and assembled a de novo reference transcriptome. In total, 60 GB of raw data were obtained, resulting in 43,531 assembled transcripts in the reference transcriptome, of which 26,525 remained after quality filtering, i.e., selecting for longest isoform length, duplicate, and redundancy removal. Then, we mapped the reads of the respective samples back to the reference transcriptome. Transcription expression patterns were highly similar among replicates, whereas patterns were clearly different between treatments (Fig. 2A).

### Differentially expressed enrichments

To estimate functional changes in expression patterns between treatments, we determined 16,473 differentially expressed genes (DEGs), of which 15,327 were found to be protein-coding. Protein-coding DEGs were used to identify specific pathways associated with either treatment. For this, protein-coding DEGs were annotated and subjected to pathway over-representation, gene set enrichment analyses, and gene ontology (GO) term enrichment analysis. As annotation of protistan genomic data is challenging, these DEGs were annotated with both, the human KOBAS database and a universal eukaryote eggNOG-mapper database, obtaining 3497 and 3577 annotated genes respectively of which 3093 were shared [28–30]. The pathway over-representation analysis revealed DEGs assigned to numerous pathways (Fig. 2B), including pathways like “Cell cycle,” “Oocyte meiosis,” and “DNA replication.” The gene set enrichment analysis showed similar results but further indicated enrichment in sulfur-related metabolism, including “Glutathione” and “Cysteine and methionine metabolism” pathways. To identify specifically upregulated DEGs between the two treatments, we then analyzed the GO term enrichment. A total of 8345 genes (of which 1929 could be annotated) were significantly upregulated in the non-starved treatment and 8128 genes (of which 1648 could be annotated) in the starved treatment. In total, 300 GO terms and 23 Kyoto Encyclopedia of Genes and Genomes (KEGG) terms with differentially expressed genes were obtained for the non-starved treatment, and 112 GO terms and 7 KEGG terms with differentially expressed genes were obtained for the starved treatment. Subsequently, to elucidate the biological significance represented by numerous GO terms, the GO terms were simplified. Fourteen and nine simplified groups were obtained for the non-starved (Fig. 2C) and starved treatment (Fig. 2D). In the non-starved treatment, upregulated simplified groups included terms such as “Mitotic,” “Cell division,” “Lamellipodium,” and “Metabolic,” indicating mitotic division, rapid growth, and pseudopodia-facilitated predation, as expected for the treatment and consistent with the observed behaviors of cells (Fig. 2C). In the starved treatment, we observed an upregulation of terms such as “Cell adhesion,” “RNA,” “Response,” and “Macropinocytosis,” supporting the expected transcriptomic response to fusion, starvation stress, and an increased communication. The upregulation of macropinocytosis-related genes might indicate a non-selective uptake of remaining environmental nutrients as a stress response [31–33].

### Differential expression of meiosis-associated transcriptions

**Fig. 3:**
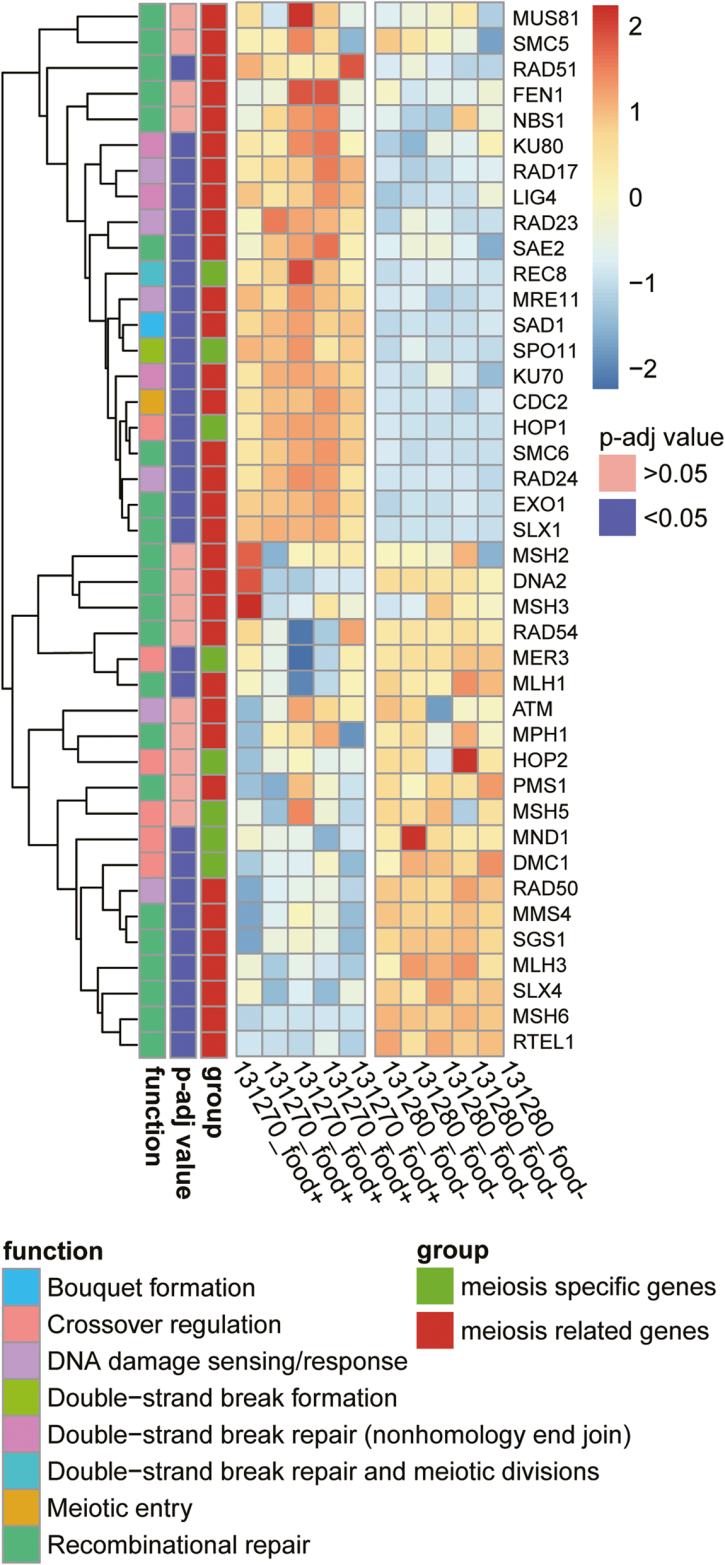
Overview of the differential expression of 41 meiosis-associated genes, their functions, assignment, and expression. Numerous genes are up- or downregulated between treatments. The left dendrogram of gene expression clustering shows the separation of the ten samples and two treatments

Lastly, genes that are typically associated with meiosis in species with sexual reproduction were identified in *F. terrestris* and analyzed for changes in their expression. A previously published list of meiosis-associated genes comprised 11 meiosis-specific and 40 meiosis-related genes [7]. Here, a total of 41 out of 51 meiosis-associated genes were detected, including eight meiosis-specific genes and 33 meiosis-related genes (Fig. 3; Additional file 1: Table S1). The ten missing transcriptions in *F. terrestris* included *MSH4*, *ZIP1*, *RED1*, *LIF1*, *REC114*, *BRCA1*, *BRCA2*, *YEN1*, *MEC1*, and *RAD52*.

Among the eight meiosis-specific genes, three (*DMC1*, *MND1*, and *MER3*) were upregulated and three (*SPO11*, *HOP1*, and *REC8*) were downregulated during starvation. The three upregulated genes function in crossover regulation. The downregulated genes, *REC8* forms the bouquet, *HOP1* regulates crossover formation, and *SPO11* forms the double-strand break during meiosis. The meiosis-specific genes *HOP2* and *MSH5* did not exhibit significant expression changes between treatments [5].

Additionally, we identified 22 meiosis-related DEGs, of which 8 were upregulated and 14 were downregulated during starvation. All upregulated genes contribute to recombinational repair, except for *RAD50*, which functions in DNA damage sensing/response. The downregulated DEGs fall into five distinct functional groups: five genes (*EXO1*, *SLX1*, *SMC6*, *RAD51*, and *SAE2*) fulfill functions in recombinational repair; four genes (*RAD17*, *RAD24*, *RAD23*, and *MRE11*) are involved in DNA damage sensing/response; three genes (*LIG4*, *KU70*, and *KU80*) are assigned to double-strand break repair (non-homologous end joining); *CDC2* indicates meiotic entry; and *SAD1* is involved in bouquet formation. Eleven meiosis-related genes did not change significantly in expression intensity between treatments [5].

## Discussion

### *F. terrestris* expresses meiosis-associated genes during starvation

We were able to determine 41 of the initially surveyed 51 meiosis genes. Twenty-eight of the 41 detected meiosis-associated genes were shown to be differentially expressed. Seventeen of these genes were downregulated during starvation. Downregulated genes include predominantly genes that are thought to be involved in five distinct meiosis-related processes: (1) double-strand break and its repair (*SPO11*, *REC8*, and the three genes *LIG4*, *KU70*, and *KU80*, both being involved in nonhomology end joining integration of DNA); (2) five genes that are reported to function in recombinational repair (*RAD51*, *SAE2*, *SLX1*, *EXO1*, and *SMC6*); (3) four genes belong to DNA damage sensing/response function, including *RAD17*, *RAD23*, *RAD24*, and *MRE11*; (4) *CDC2* indicating “Meiotic entry”; and (5) *HOP1* regulates crossover. Specifically, *SPO11* produces DNA double-strand breaks (DSBs), which are necessary for meiotic recombination [34]. According to studies in yeast, the expression of *SPO11* peaks at meiotic entry and then gradually declines [35]. Starving yeast was shown to induce genome-wide meiotic *SPO11*-dependent double-strand breaks, and then return to mitotic growth when food was resupplied indicating a temporal separation and termination of initiated meiosis, a process known as “Return To Growth” (RTG) [36, 37]. Considering that fused *F. terrestris* can separate when food is resupplied (Fig. 1B, right), a temporal separation of meiosis and an RTG-like process in protists like *F. terrestris* is thinkable, potentially explaining the mixed response of meiosis-associated genes in our analysis.

Most upregulated meiosis-associated genes during fusion of *F. terrestris* are known to be involved in crossover regulation (*DMC1*, *MER3*, and *MND1*) and recombinational repair (*MLH1*, *MMS4*, *MSH6*, *MLH3*, *SGS1*, *SLX4*, and *RTEL*). Upregulated *RAD50* is related to DNA damage sensing/response. Among the upregulated genes, including the equally expressed *HOP2*, *DMC1* is expected to be crucial for entering meiosis as it promotes the formation of strand-invasion products (D-loops) between homologous molecules [27]. *MND1* and *HOP2* stabilize the binding of *DMC1* and DNA [38–40]. Thus, their simultaneous activity suggests active recombination between homologies. The last upregulated meiosis-specific gene, the *MER3* helicase is a member of the ZMM protein group (also known as the synapsis initiation complex = SIC), facilitating the formation of the majority of crossovers during meiosis [41, 42]. Based on the differential expression of said genes, we hypothesize that *F. terrestris* undergoes double-strand breaks and homologous recombination during fusion.

We were not able to detect the expression of three meiosis-specific transcriptions (*MSH4*, *ZIP1*, and *RED1*) and seven meiosis-related transcriptions (*LIF1*, *REC114*, *BRCA1*, *BRCA2*, *YEN1*, *MEC1*, and *RAD52*), indicating their absence. These transcriptions may either not have been expressed during our experiment or may have been lost in *F. terrestris*. Many members of the SAR clade seem to miss some or all of those genes, such as *MSH4* [5, 43]. *ZIP1* and *RED1* are both involved in the synaptonemal complex, which is not strictly needed for meiosis in many taxa [43]. The common absence of these genes from transcriptomic or genomic data in the SAR clade might indicate a loss of those genes in its common ancestor (consisting of the Stramenopiles, Alveolata, and Rhizaria, see Fig. 1A). However, the absence of meiosis-associated genes does not necessarily confirm an absence of meiosis. For example, *Caenorhabditis elegans* and *Drosophila melanogaster* are missing a whole series of meiosis-related genes and evidently undergo meiosis nonetheless as these taxa exclusively using crossover pathway 1 [44, 45]. The same applies to the protistan Alveolata (in SAR group) for which there is plenty of evidence for sex, but an absence of numerous meiosis-associated genes [5].

### Sulfur availability may trigger fusion and recombination in *F. terrestris*

Under starvation, the amoeba *Dictyostelium discoideum* (Amoebozoa, Fig. 1A) shifts from a unicellular to a multicellular life history stage and forms sexually derived spores [46, 47]. During extensive research on this model organism, it was found that harmful reactive oxygen species (ROS) form under starvation. As a response, the amino acid cysteine is sequestered in the antioxidant glutathione. Altogether, this links sulfur availability to fusion and sex in this protist [48]. Although only distantly related, we found the downregulation of the pathways “Cysteine and methionine metabolism” and “Glutathione metabolism” indicating this to be a conserved eukaryotic response. Even in bacteria, sulfur metabolism regulates cell cycle progression via shifts in glutathione levels [49].

## Conclusions

The detection of sexual processes in protists is generally quite challenging because of their small size, restricted culture conditions, and limited access to genetic material. A number of studies interpreted the presence of conserved meiosis genes as evidence for sex [50]. As discussed, such conserved genes are often pleiotropic and meiosis can be present in asexual organisms [21–23, 51]. Consequently, the presence of meiosis genes alone is of limited reliability for detecting meiosis and sex. Here, to alleviate some of the mentioned challenges, we induced fusion in *F. terrestris* and analyzed the transcriptional response with a specific focus on meiosis-associated genes and processes. Based on the findings here, the genes that likely regulate crossover and repair recombination are upregulated, so meiosis might be active, but temporal dynamics and RTG-like processes could confound meiosis-associated expression dynamic patterns which cannot be ruled out. Ultimately, to clearly elucidate the occurrence and role of meiosis, genetic recombination, and sexual processes in Rhizaria, further studies are needed analyzing, e.g., fine-scale cytological processes during fusion and/or population genetic outcomes of sex such as allele sharing among individuals. It is important to note that while meiosis, genetic recombination, and sexual reproduction are often associated, they are not universally linked across all eukaryotic groups. For instance, in ciliates, meiosis and genetic exchange occur without being directly tied to reproduction. This highlights the need for careful distinction during the investigation of these processes in diverse eukaryotic lineages, including Rhizaria. *Fisculla terrestris* is a well-suiting candidate to further investigate putative sexual reproduction in distant relatives of animals and plants. Cultures of *F. terrestris* can be easily established and maintained, fusion can be easily induced, and its slow and transparent cells facilitate microscopical investigation.

## Methods

### Experimental setup and transcriptome sequencing

*F. terrestris* was cultured in Waris-H+Si (McFadden & Melkonian) with *Saccharomyces cerevisiae* as food in 80 ml batch cultures. Under these culture conditions, the highest density of *F. terrestris* cells was expected after 5 days. We, therefore, harvested five replicates for transcriptome sequencing after 4 days of culture growth when food was declining in abundance, and another set of five replicates was harvested after an additional 3 days of growth, when food was depleted and a large number of cells were fused or dormant. RNA was extracted with the Qiagen RNeasy plant mini kit. For this, the culture flasks were vigorously shaken to detach amoeba cells from the plastic surface, and approx. 80 ml of each culture was filtered with an 8-µm pore size filter to maximize the density of *F. terrestris* while minimizing contamination by environmental bacteria and *S. cerevisiae*. The filter was given into a 1.5-ml Eppendorf tube with 1 ml of ice-cold Soerensen buffer and vortexed vigorously to detach the cells from the filter. Subsequently, the Eppendorf tube was centrifuged at 1000 rpm for 5 min at 4 °C and the filter was removed without disturbing the pellet. The Eppendorf tube was centrifuged at 1000 rpm for 2 min and 4 °C to firm the pellet. The Soerensen buffer was discarded and replaced by 1 ml of clean new buffer. The tube was centrifuged at 1000 rpm for 5 min and 4 °C to firm the pellet. The Soerensen buffer was discarded and replaced by 170 µl ice-cold RLN buffer. The tube was vortexed, and 450 µl RLT buffer of the Qiagen RNeasy plant mini kit was added and mixed by vortexing. The tubes were kept in liquid nitrogen until following the rest of the manual’s instructions for the Qiagen RNeasy plant mini kit. Poly-A selection, cDNA synthesis, paired-end library preparation, and subsequent Illumina NovaSeq 6000 sequencing were conducted in the West German Genome Center.

### Reference transcription assembly

Sequence reads were trimmed with TrimGalore (version v0.6.5, https://github.com/FelixKrueger/TrimGalore) before assembly with Trinity (version v2.1.1) [52]. We used the default parameter for assembly and then used Trinity’s “get_longest_isoform_seq_per_trinity_gene.pl” to select for the longest transcripts. The CD-HIT (version v4.8.1) [53] was employed to select the unique transcripts according to a similarity criterion of 90% and reads with a length of <300 bp were removed by Seqkit (version 0.14.0) [54]. Translation of transcripts was performed by Transdecoder (https://github.com/TransDecoder/TransDecoder/wiki; last accessed November 14, 2018) to establish the protein data set used for further analyses.

### Contamination removal and quality assessment

To identify and remove putative contamination by the food organism *Saccharomyces cerevisiae*, we downloaded its genome and related protein sequences (ASM308665v1). We used Minimap2 (version v2.1) [55] and Blastp [56] to screen for the presence of potential yeast sequences. In addition, we used the BlobTools2 kit (version v2.3.3) [57] to check the results made by Busco (version 5.0.0) [58] and Blastp. Most of the transcripts (29M/30M) were no-hit in the nt database and the Busco results showed a completeness of 77.3% against the eukaryota_obd10 database.

### Differentially expressed genes detection

To identify and quantify the DEGs, we used RSEM (version v1.3.3) [59] to map the filtered reads to the reference transcriptome with parameters “--est_method RSEM --aln_method bowtie2 --trinity_mode --prep_reference.” DEGs were determined with DeSeq2 (version v. 1.10.1) [60]. Transcripts with <50 reads among all samples were discarded and we only kept DEGs with an adjusted *p* value of less than 0.05.

### Annotation and enrichment of DEGs

The longest resulting protein sequences were used for annotation. DEGs were annotated with KOBAS (version v. 3.0) [29] and its human database and eggNOG-mapper (version v2.1.6) [30]. Subsequently, the Kyoto Encyclopedia of Genes and Genomes (KEGG) pathways and gene ontology (GO) enrichment analysis and gene set enrichment analysis (GSEA) were carried out using clusterProfiler (version v. 3.15) [61]. The protein sequences derived from DEGs were submitted to the human database, and hypergeometric test/Fisher’s exact test were used to calculate the corrected *p* values.

### Meiosis-associated genes Blast

Lastly, we compared our genes to 11 meiosis-specific and 40 meiosis-related genes published before [5, 50]. For this, we aligned these sequences to our protein database. Aligned sequences with an *e* value <10E^−4^ were kept and then manually curated.

## Supplementary Information

Additional file 1: Table S1 DESeq2 expression level of the 41 DEGs showed the raw expression.

## Data Availability

The raw data is available at NCBI Bioproject PRJNA1108686 [62].

## Abbreviations

DEGs: Differentially expressed genes
GO: Gene ontology
KEGG: Kyoto Encyclopedia of Genes and Genomes
DSBs: Double-strand breaks
RTG: Return to growth
SIC: Synapsis initiation complex
ROS: Reactive oxygen species
GSEA: Gene set enrichment analysis
PCA: Principal component analysis
